# When Should the File Be Used?

**Published:** 1869-04

**Authors:** W. F. Morrill


### WHEN SHOULD THE FILE BE USED?

BY W. F. MORRILL.

[Read before the Wabash Dental Association, March 24, 1869.]

The practice of using the file on the teeth has been common more than one hundred years. The pioneer Dentists knew little beyond this method in preserving the teeth. Rude and ill-contrived as their investments were, they were compelled to have more space in which to perform their operations than we of a later day. Nicer perceptions of the file's uses have been revealed unto us, and the former excesses, to which many were guilty, have either been abandoned or they are confined to those who revere antiquated habits and customs.

The form of this question—when should the file be used?—infers that at some time or another there are pathological indications which would justify us in its use; that the objects we have in view can not be so well secured as by employing the file. It is true, however, we have among us those who presume to have arrived at that par excellent point of practice where the file becomes obsolete, while other poor mortals, not so blest, are left content to drift along in a custom which has the must of age upon it. Of the former, ignoring the use of the file, I have no acquaintance, nor can I speak with confidence of their successes.

As auxilliary to the purposes of the file, I may mention the large variety and forms of chisels which are now introduced to the profession as possessing great advantages. The separations between teeth can be much more quickly done, the breaking down of projecting enamel walls, and otherwise facilitating our operations in these respects by the aid of chisels than with the use of files. But with all this speed in removing tooth substance, it does not necessarily follow that we shall discard the file. It still has its separate duties to perform. The smooth and regular surfaces, the forms and shapes we desire the teeth to assume as initiatory to filling, require the file. The slight, but necessary, service it does around the cavity's edges just previous to filling, we are beginning to know how important it is.

Of the many abuses of the file there are just grounds of complaint. As an evidence of mischief or distrust the discussion now provoked in this direction is ample testimony. The liberal and, in some instances, the unsparing use of the file should arouse us to a sense of inquiry whether our present practice be sound and correct. We can not disguise the fact that thousands of teeth pay their annual tribute to this sacrificial alter. To destroy one-third or one-half of the tooth substance to preserve the balance, is certainly very primitive practice. The numerous bicuspids, molars and laterals, having so much of the tooth tissue wasted, is not flattering nor in keeping with the enlightened progress of our science. By this destructive method, the train of evils afflicting the patients becomes troublesome; particles of food force their way up between the approximal surfaces of the teeth; the gum and investing membrane of the teeth become irritated, which occasions much inconvenience. Added to these evils, we have the loss of the masticating surfaces, together with the symmetry and beauty of the teeth. Moreover, along the margins of cavities filled, and the dentine tissue left unprotected by a flange of gold extending over it, we find streaks of caries making their appearance. Does this practice warrant a continuance, or does it more properly belong to the Chinese or East Indies, rather than to the art and science of American Dentistry?

The time to use the file depends on *the end we have in view*, *the age of the patient, and the physical characteristics of the teeth.* The indications for the employment of the file are varied and dissimilar. We can scarcely settle upon a fixed rule or method by which to be governed in each case. If we did, unhappy results would surely follow. Among the chief objects to be secured by the file is room for further

Dental operations. This is now far better attained by the introduction of wedges.

The arrest of caries forms no inconsiderable share of this instrument's use. For this purpose, especially in the incipient stages, recourse to the file is indispensable. It is not uncommon to hear Dentists talk quite fluently of the freedom of it on deciduous teeth. It is barely possible their class of patients at this tender age may be more tractable than falls to the lot of others less fortunate, who are unable to speak so favorably of their successes. But the composure necessary for children passing this ordeal is rarely found, and the benefits derived in this direction can not be very many. The green stain deposits on temporary and permanent teeth, which usually require removal, can be done quite as well by employing corundum points and burnishers. The most critical time for our decision when to employ the file may be dated between twelve and sixteen years. At these respective periods with regard to how much tooth substance shall be destroyed, in order to preserve the harmony and beauty of dentures, demands the exercise of caution and sound judgment. If predisposing signs of caries are manifest, affording apprehension for the future, there should be no hesitancy about the use of file; nor should there be any want of alacrity in its use when dentures are crowded by approximal surfaces of teeth overlapping each other, and no reasonable hope of further expansion of the maxillary bones, either naturally or artificially. That good results are certain to follow at these periods experience clearly demonstrates, and the logic of events can not show to the contrary. Wherever superficial caries prevails the file is serviceable. What the chisel is in the hands of a sculptor, so is the file in the hands of a Dental Artist.

Culture and experience are reformatory in the use of this valued instrument. We can think of it in the hands of an incompetent person as a weapon of mischief, and in the hands of an intelligent practitioner as one of incomparable value.

When to exercise its freedom and when to be sparing of its use, requires observations ripened by more experience than is usually possessed by the many. In a single paper it would be difficult to even hint at the precise dates when the file should be used, and I will not attempt the task. If, however, I have awakened your minds to a higher conception of this important branch of operative Dentistry, I will achieve all I intended.

